# Inverse Association between Hepatitis B Virus Infection and Fatty Liver Disease: A Large-Scale Study in Populations Seeking for Check-Up

**DOI:** 10.1371/journal.pone.0072049

**Published:** 2013-08-22

**Authors:** Yuan-Lung Cheng, Yuan-Jen Wang, Wei-Yu Kao, Ping-Hsien Chen, Teh-Ia Huo, Yi-Hsiang Huang, Keng-Hsin Lan, Chien-Wei Su, Wan-Leong Chan, Han-Chieh Lin, Fa-Yauh Lee, Jaw-Ching Wu

**Affiliations:** 1 Division of Gastroenterology, Department of Medicine, Taipei Veterans General Hospital, Taipei, Taiwan; 2 Division of Healthcare and Services, Department of Medicine, Taipei Veterans General Hospital, Taipei, Taiwan; 3 Faculty of Medicine, School of Medicine, National Yang-Ming University, Taipei, Taiwan; 4 Division of Gastroenterology and Hepatology, Department of Internal Medicine, Taipei Medical University Hospital, Taipei, Taiwan; 5 Endoscopy Center for Diagnosis and Treatment, Taipei Veterans General Hospital, Taipei, Taiwan; 6 Department and Institute of Pharmacology, National Yang-Ming University, Taipei, Taiwan; 7 Institute of Clinical Medicine, School of Medicine, National Yang-Ming University, Taipei, Taiwan; 8 Department of Medical Research and Education, Taipei Veterans General Hospital, Taipei, Taiwan; National Institute for Viral Disease Control and Prevention, CDC, China

## Abstract

**Background:**

Although many studies have attempted to clarify the association between hepatitis B virus (HBV) infection and fatty liver disease, no prior studies have emphasized the relationship of HBV and fatty liver regarding different demographics of age and body mass index (BMI).

**Aim:**

To investigate the correlation of HBV and fatty liver in the different demographics of age and BMI.

**Methods:**

We enrolled consecutive subjects who had received health check-up services at the Taipei Veterans General Hospital from 2002 to 2009 and ultrasonography was used to diagnose fatty liver according to the practice guidelines of the American Gastroenterological Association.

**Results:**

Among the 33,439 subjects enrolled in this study, fatty liver was diagnosed in 43.9% of the population and 38.9% of patients with chronic HBV infection. Multivariate analysis showed that BMI, age, waist circumference, systolic blood pressure, fasting glucose, cholesterol, alanine aminotransferase (ALT) levels, and platelet counts were positively associated, while hepatitis B surface antigen (HBsAg) positivity was inversely associated with fatty liver, especially for subjects with BMI>22.4 kg/m^2^ and age>50 years. On the contrary, HBV infection was positively correlated with the presence of elevated serum ALT levels in subjects with fatty liver disease regardless of their age and BMI.

**Conclusions:**

Metabolic factors are important determinants for the prevalence of fatty liver. Patients with HBV infection were inversely associated with fatty liver disease than the general population, especially in older and obese patients. Furthermore, metabolic factors and HBV infection were associated with elevated serum ALT levels in fatty liver disease.

## Introduction

Nonalcoholic fatty liver disease (NAFLD) is the most common cause of abnormal liver biochemistry tests in the world, both in high and low endemic countries for viral hepatitis [1]–[3]. With the different study populations and diagnosing modalities, the prevalence rate of NAFLD has been reported to be between 10–35% in the United States and it is correlated to metabolic factors, such as central obesity, insulin resistance, arterial hypertension, and hypertriglyceridemia [4].

With the increasing prevalence of the Western dietary pattern, the prevalence of NAFLD also continues to rise in Asian countries. A Korean study reported a NAFLD prevalence rate was 51% in 589 potential liver donors and the risk factors included older age, obesity and hypertriglyceridemia [5]. One population-based study from China revealed that the NAFLD is present in 23.3% of the study group and it is associated with higher serum alanine aminotransferase (ALT), triglyceride (TG) and fasting glucose levels [6]. However, the correlation between hepatitis B virus (HBV) infection and fatty liver disease has not been fully elucidated [7]–[13]. HBV X protein has been proven to influence lipogenic genes, such as sterol regulatory element binding protein-1c, fatty acid synthase, and peroxisome proliferator-activated receptor [14]. Nevertheless, several studies demonstrated chronic HBV infection was not associated with hepatic steatosis and insulin resistance, and hepatic steatosis in HBV infection is associated with metabolic but not viral factors [9], [12], [15]–[17].

Although many studies have tried to clarify the association between HBV infection and fatty liver disease [7]–[9], [11], [12], [16]–[18], no previous studies comprehensively emphasized on the relationship of HBV and fatty liver in the different demographics of age and body mass index (BMI). This large-scale cross-sectional study in Taiwan aimed to investigate the correlation of chronic hepatitis B virus infection and fatty liver in Taiwan.

## Materials and Methods

### Study Population

**Figure 1:**
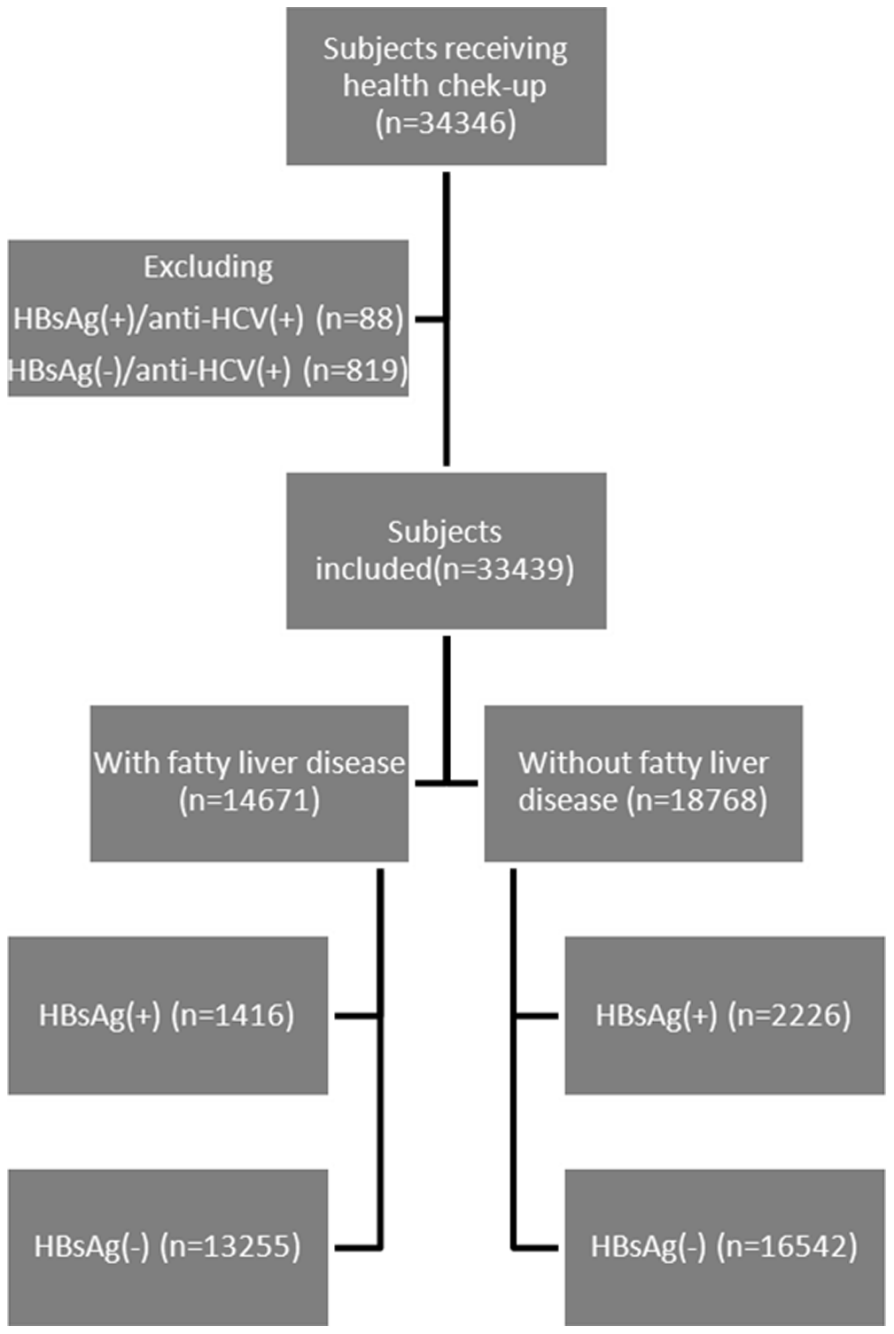
The flow of subjects in the study.

There were 34,346 subjects receiving health check-up services at the Taipei Veterans General Hospital from 2002 to 2009 [2], [19]. As shown in **Figure 1**, those who had chronic HCV infection were excluded and the remaining 33,439 subjects were included in the analysis. All of them underwent complete clinical evaluation, laboratory examination and abdominal sonography. BMI was the result of the division of the body weight (in kilograms) by the square body height (in meters). We adopted a normal body mass index (BMI) was between 17.5 and 22.4 kg/m^2^, an overweight BMI between 22.5 and 24.9 kg/m^2^, and an obese BMI higher than 25 kg/m^2^ [20]. Blood pressure (BP) was measured after the subjects had been seated for more than 5 min. The means of three consecutive readings were recorded as systolic and diastolic BP with a difference in systolic BP<10 mmHg. Ultrasonography with Aloka SSD 4000 (Aloka, Tokyo, Japan), Aloka SSD 5000 (Aloka) or Philips HD 15 (Philips, Bothell, WA, USA) were used to diagnose fatty liver by the practice guideline of the American Gastroenterological Association [1]. The study followed the standards of the Declaration of Helsinki and has been approved by the Institutional Review Board of the Taipei Veterans General Hospital (2011-08-010IC). As the dataset used in this study is consisted of de-identified data from a retrospective cohort, the written informed consents from the subjects who receiving physical check-up services were waived by the approval of the IRB.

### Biochemical and Serologic Markers

Venous blood samples were collected after an overnight fast. Radio-immunoassay (Abbott Laboratories, North Chicago, IL, USA) was used to test serum HBV surface antigen (HBsAg) and second-generation enzyme immunoassay (Abbott Laboratories) was used to test antibody to hepatitis C virus (anti-HCV). The Roche/Hitachi Modular Analytics System (Roche Diagnostics GmbH, Mannheim, Germany) was utilized to measure serum biochemical markers. The reference limits of these tests were as follows: ALT level, 40 IU/L; gamma-glutamyltransferase (GGT) level 51 IU/L; total cholesterol level, 200 mg/dL; high-density lipoprotein-cholesterol (HDL) level, 40 mg/dL for men and 50 mg/dL for women; low-density lipoprotein-cholesterol (LDL) level, 130 mg/dL; TG level, 150 mg/dL; fasting glucose level, 100 mg/dL; and 2-h post-load plasma glucose level, 150 mg/dL. Fatty liver index (FLI) was calculated as the following formula: FLI = (e ^0.953*loge (TG) +0.139*BMI +0.718*loge (GGT) +0.053*waist circumference - 15.745^)/(1+ e ^0.953*loge (TG) +0.139*BMI +0.718*loge (GGT) +0.053*waist circumference - 15.745^) * 100 [21].

### Statistical Analysis

The population was analyzed by stratification of HBV infection and fatty liver disease. Subsequently, the different characteristics of subjects with and without fatty liver or HBV infection were analyzed, respectively. The population was then stratified with age, BMI and HBV status.

Pearson chi-squared and Student t-test analysis was performed to compare categorical and continuous variables with two samples, respectively. Variables with statistical significance (P<0.05) or proximate to it (P<0.1) in univariate analysis were included in multivariate analysis by logistic regression model with the forward stepwise selection procedure.

## Results

### Subjects’ Characteristics Stratified by HBV Infection and Fatty Liver

**Figure 2:**
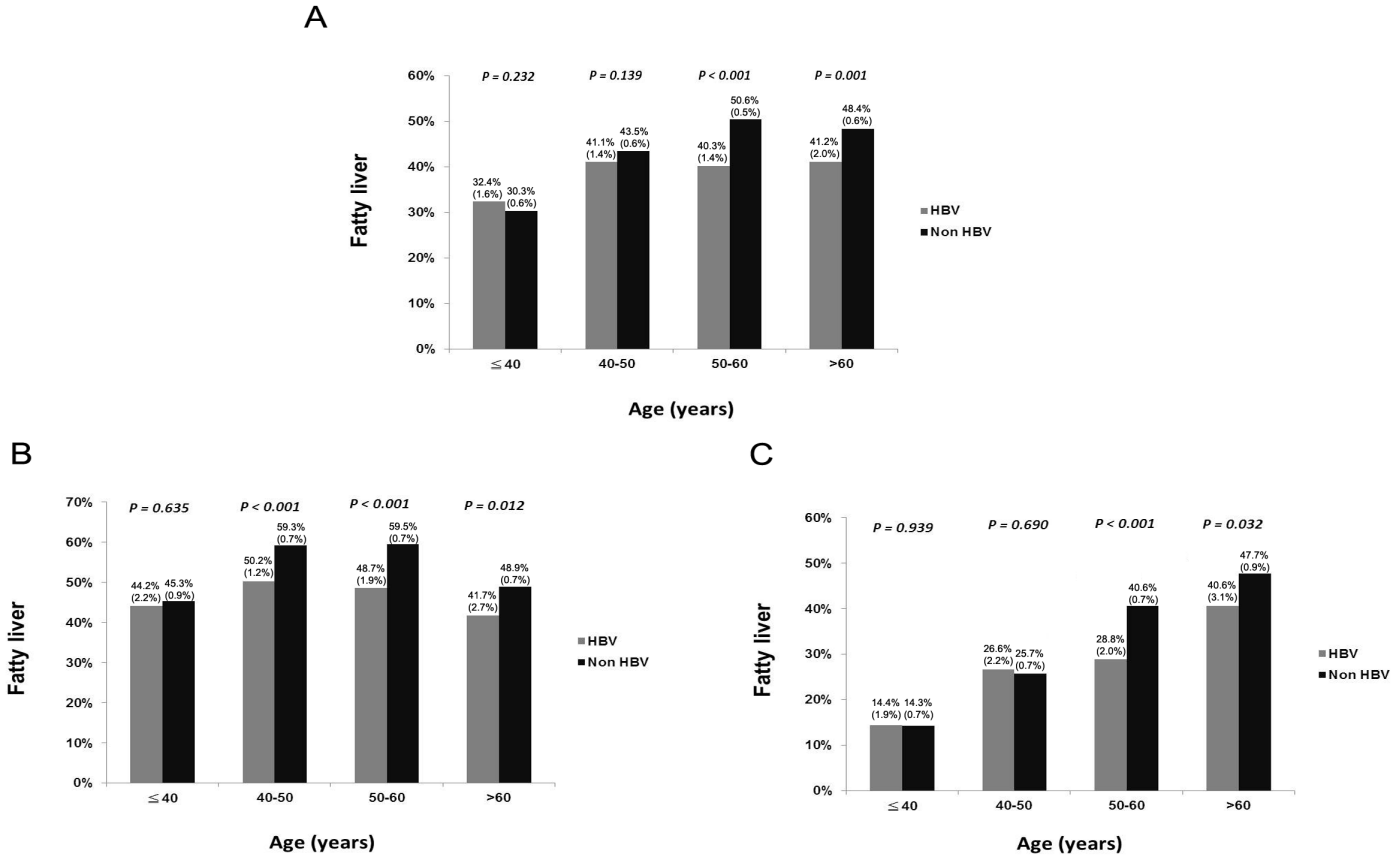
Age distribution of fatty liver stratified by HBV status (A) all subjects, (B) males and (C) females. The prevalence rates of fatty liver in all subgroups are expressed as prevalence rate (standard error).

The demographic data of all subjects are shown in **Table 1**. The mean age was 51.9 years, with a male prevalence of 54.6% and fatty liver was diagnosed in 43.9% of the whole population by ultrasonography. Patients with HBV infection were younger in age, moremales, with higher levels of ALT and AST levels, lower systolic blood pressure (SBP), lower levels of cholesterol, LDL, TG, glucose levels, and platelet counts (all of the *P*<0.001). Fatty liver presented in 38.9% of HBV patients, lower than the 44.5% in non-HBV subjects (*P*<0.001). **(** **Table 1** and **Figure 2** **).**

**Table 1:** Factors associated with HBV and non-HBV subjects.

	ALL (n = 33439)	HBV(+) (n = 3642)	HBV(−) (n = 29797)	P value
**BMI, kg/m^2^** *	23.81±3.56	22.77±3.38	23.82±3.56	0.434
**Age, years** *	51.9±13.1	49.5±11.5	52.2±13.3	<0.001
**Sex (M/F) (%)**	18257/15182 (54.6%/45.4%)	2159/1483 (59.3%/40.7%)	18681/15665 (54.4%/45.6%)	<0.001
**WC, cm** *	83.7±10.2	83.4±9.9	83.8±10.3	0.051
**SBP, mmHg** *	124.1±18.5	122.5±17.8	124.3±18.6	<0.001
**DBP, mmHg** *	77.5±14.4	77.3±15.7	77.5±14.3	0.399
**Fasting glucose, mg/dL** *	95.4±24.6	94.2±23.4	95.5±24.8	<0.001
**Cholesterol, mg/dL** *	198.6±37.0	193.5±36.8	199.2±37.0	<0.001
**HDL, mg/dL** *	53.7±15.1	53.7±15.4	53.7±15.0	0.747
**LDL, mg/dL** *	124.9±32.9	121.5±32.7	125.3±32.9	<0.001
**TG, mg/dL** *	128.9±87.4	116.6±80.2	130.4±88.1	<0.001
**AST, IU/L** *	23.7±15.0	28.8±24.9	23.1±13.2	<0.001
**ALT, IU/L** *	28.2±26.4	37.7±47.6	27.0±22.2	<0.001
**GGT, IU/L** *	24.7±36.5	24.3±33.2	24.8±36.8	0.504
**Platelet, 1000/mm^3^** *	247.6±60.7	229.1±60.6	249.8±60.3	<0.001
**Fatty liver (yes/no) (%)**	14671/18768 (43.9/56.1)	1416/2226 (38.9%/61.1%)	13255/16542 (44.5%/55.5%)	<0.001
**FLI** *	26.96±24.03	24.72±22.67	27.24±14.18	<0.001

HBV, hepatitis B virus; BMI, body mass index; SD, standard deviation; M, male; F, female; WC, waist circumference; SBP, systolic blood pressure; DBP, diastolic blood pressure; HDL, high-density lipoprotein; LDL, low-density lipoprotein; TG, triglyceride; AST, aspartate aminotransferase; ALT, alanine aminotransferase; GGT, gamma-glutamyltransferase; FLI, fatty liver index.

* Expressed as mean ± standard deviation.

As shown in **Table 2**, compared to subjects without fatty liver, those with fatty liver were older in age, more males, and had higher BMI, larger waist circumference (WC), higher SBP, higher levels of glucose, cholesterol, LDL, TG, ALT, GGT, platelet counts, FLI, but lower HDL levels (all of the *P*<0.001) by univariate analysis.

**Table 2:** Comparison of demographic characteristics between subjects with and without fatty liver.

	Without fatty liver (n = 18768 )	With fatty liver (n = 14671)	P value
**BMI, kg/m^2^** *	22.35±2.90	25.68±3.46	<0.001
**Age, years** *	50.6±13.9	53.6±11.9	<0.001
**Sex (M/F) (%)**	8531/10237(45.3%/54.7%)	9726/4945(66.1%/33.9%)	<0.001
**WC, cm** *	79.6±8.9	89.1±9.3	<0.001
**SBP, mmHg** *	121.0±18.4	128.0±18.0	<0.001
**Fasting glucose, mg/dL** *	90.6±18.9	101.5±29.4	<0.001
**Cholesterol, mg/dL** *	194.1±36.0	204.2±37.6	<0.001
**HDL, mg/dL** *	58.1±15.5	48.0±12.4	<0.001
**LDL, mg/dL** *	120.2±31.8	130.9±33.3	<0.001
**TG, mg/dL** *	100.5±57.0	165.3±104.4	<0.001
**ALT, IU/L** *	22.7±24.1	35.2±27.4	<0.001
**GGT, IU/L** *	20.1±33.7	30.7±38.9	<0.001
**Platelet, 1000/mm^3^** *	245.1±61.5	250.8±60.7	<0.001
**HBsAg** **(positive/negative) (%)**	2226/16542(11.9%/88.1%)	1416/13255(10.7%/89.3%)	<0.001
**FLI** *	15.64±16.31	41.45±24.54	<0.001

BMI, body mass index; SD, standard deviation; M, male; F, female; WC, waist circumference; SBP, systolic blood pressure; HDL, high-density lipoprotein; LDL, low-density lipoprotein; TG, triglyceride; ALT, alanine aminotransferase; GGT, gamma-glutamyltransferase; HBsAg, hepatitis B surface antigen; FLI, fatty liver index.

* Expressed as mean ± standard deviation.

For patients with HBV infections, the demographic characteristics between those with and without fatty liver were compared in **Table 3**. Similarly, subjects with fatty liver were older in age, more males, and had higher BMI, larger WC, higher SBP, higher levels of fasting glucose, cholesterol, LDL, TG, ALT, GGT, platelet counts, FLI, but lower HDL levels by univariate analysis.

**Table 3:** Comparison of demographic characteristics between HBV subjects with and without fatty liver.

	Without fatty liver (n = 2226 )	With fatty liver (n = 1416)	P value
**BMI, kg/m^2^** *	22.57±2.86	25.65±3.28	<0.001
**Age, years** *	49.0±11.8	50.3±10.9	0.001
**Sex (M/F) (%)**	1143/1083 (51.3%/48.7%)	1016/400 (71.8%/28.2%)	<0.001
**WC, cm** *	79.9±8.8	89.0±9.0	<0.001
**SBP, mmHg** *	120.0±17.4	126.3±17.8	<0.001
**Fasting glucose, mg/dL** *	90.1±17.3	100.4±29.5	<0.001
**Cholesterol, mg/dL** *	191.5±36.0	196.8±37.6	<0.001
**HDL, mg/dL** *	57.7±15.6	47.5±12.9	<0.001
**LDL, mg/dL** *	118.5±32.0	126.3±33.3	<0.001
**TG, mg/dL** *	96.7±55.2	147.7±100.8	<0.001
**ALT, IU/L** *	33.9±50.7	43.7±41.5	<0.001
**GGT, IU/L** *	21.0±32.5	29.6±34.7	<0.001
**Platelet, 1000/mm^3^** *	226.0±62.6	234.1±57.1	<0.001
**FLI** *	15.87±16.56	38.63±23.98	<0.001

BMI, body mass index; SD, standard deviation; M, male; F, female; WC, waist circumference; SBP, systolic blood pressure; HDL, high-density lipoprotein; LDL, low-density lipoprotein; TG, triglyceride; ALT, alanine aminotransferase; GGT, gamma-glutamyltransferase; HBsAg, hepatitis B surface antigen; FLI, fatty liver index.

* Expressed as mean ± standard deviation.

### Factors Associated with Fatty Liver Stratified by HBV, BMI and Age

By multivariate analysis, higher BMI, older age, higher WC, SBP, fasting glucose, cholesterol, and ALT levels, lower HDL levels, higher platelet count and HBsAg negativity were associated with fatty liver. (Table 4) For subjects with HBV infection, metabolic factors, including BMI, WC, fasting glucose, cholesterol, HDL, ALT and platelet count were correlated with fatty liver.

**Table 4:** Factors associated with fatty liver in different populations by multivariate analysis.

	Odds ratio	95% confidence interval	P
**All subjects**			
**BMI (per kg/m^2^)**	1.209	1.191–1.227	<0.001
**Age (per years)**	1.004	1.002–1.006	0.001
**WC (per cm)**	1.036	1.031–1.042	<0.001
**SBP (per mmHg)**	1.002	1.000–1.003	0.040
**Fasting glucose (per mg/dL)**	1.010	1.009–1.011	<0.001
**Cholesterol (per mg/dL)**	1.008	1.007–1.009	<0.001
**HDL (per mg/dL)**	0.969	0.967–0.971	<0.001
**ALT (per U/L)**	1.018	1.016–1.020	<0.001
**Platelet (per/mm^3^)**	1.002	1.002–1.003	<0.001
**HBsAg**	0.700	0.642–0.764	<0.001
**For subjects with HBV infection**			
**BMI (per kg/m^2^)**	1.161	1.110–1.214	<0.001
**WC (per cm)**	1.055	1.038–1.072	<0.001
**Fasting glucose (per mg/dL)**	1.013	1.009–1.017	<0.001
**Cholesterol (per mg/dL)**	1.006	1.003–1.008	<0.001
**HDL (per mg/dL)**	0.973	0.967–0.979	<0.001
**ALT (per IU/L)**	1.003	1.001–1.005	0.003
**Platelet (per/mm^3^)**	1.003	1.002–1.004	<0.001
**For subjects with BMI≤22.4 kg/m^2^**			
**Age (per years)**	1.005	1.001–1.009	0.02
**WC (per cm)**	1.071	1.062–1.080	<0.001
**Fasting glucose (per mg/dL)**	1.010	1.008–1.012	<0.001
**Cholesterol (per mg/dL)**	1.006	1.005–1.008	<0.001
**HDL (per mg/dL)**	0.977	0.973–0.981	<0.001
**ALT (per IU/L)**	1.007	1.005–1.009	<0.001
**Platelet (per/mm^3^)**	1.002	1.001–1.003	0.001
**For subjects with BMI>22.4 kg/m^2^**			
**Male gender**	1.193	1.111–1.282	<0.001
**WC (per cm)**	1.068	1.063–1.073	<0.001
**SBP (per mmHg)**	1.003	1.002–1.005	<0.001
**Fasting glucose (per mg/dL)**	1.010	1.009–1.012	<0.001
**Cholesterol (per mg/dL)**	1.008	1.007–1.009	<0.001
**HDL (per mg/dL)**	0.964	0.961–0.966	<0.001
**ALT (per IU/L)**	1.025	1.023–1.027	<0.001
**Platelet (per/mm^3^)**	1.003	1.002–1.003	<0.001
**HBsAg positivity**	0.655	0.593–0.723	<0.001
**For subjects with age≦50 years**			
**BMI (per kg/m^2^)**	1.176	1.147–1.204	<0.001
**Male gender**	0.817	0.736–0.907	<0.001
**WC (per cm)**	1.048	1.038–1.057	<0.001
**SBP (per mmHg)**	1.004	1.001–1.007	0.009
**Fasting glucose (per mg/dL)**	1.013	1.010–1.016	<0.001
**Cholesterol (per mg/dL)**	1.008	1.007–1.009	<0.001
**HDL (per mg/dL)**	0.972	0.968–0.976	<0.001
**ALT (per IU/L)**	1.011	1.009–1.014	<0.001
**GGT (per IU/L)**	1.003	1.001–1.004	<0.001
**Platelet (per/mm^3^)**	1.003	1.002–1.003	<0.001
**For subjects with age >50 years**			
**BMI (per kg/m^2^)**	1.225	1.202–1.249	<0.001
**Male gender**	1.115	1.032–1.205	0.006
**WC (per cm)**	1.030	1.023–1.036	<0.001
**Fasting glucose (per mg/dL)**	1.009	1.008–1.011	<0.001
**Cholesterol (per mg/dL)**	1.007	1.006–1.008	<0.001
**HDL (per mg/dL)**	0.967	0.964–0.970	<0.001
**ALT (per IU/L)**	1.022	1.020–1.025	<0.001
**Platelet (per/mm^3^)**	1.003	1.002–1.003	<0.001
**HBsAg positivity**	0.624	0.553–0.703	<0.001

BMI, body mass index; WC, waist circumference; SBP, systolic blood pressure; HDL, high-density lipoprotein; ALT, alanine aminotransferase; GGT, gamma-glutamyltransferase; HBsAg, hepatitis B surface antigen.

**Figure 3:**
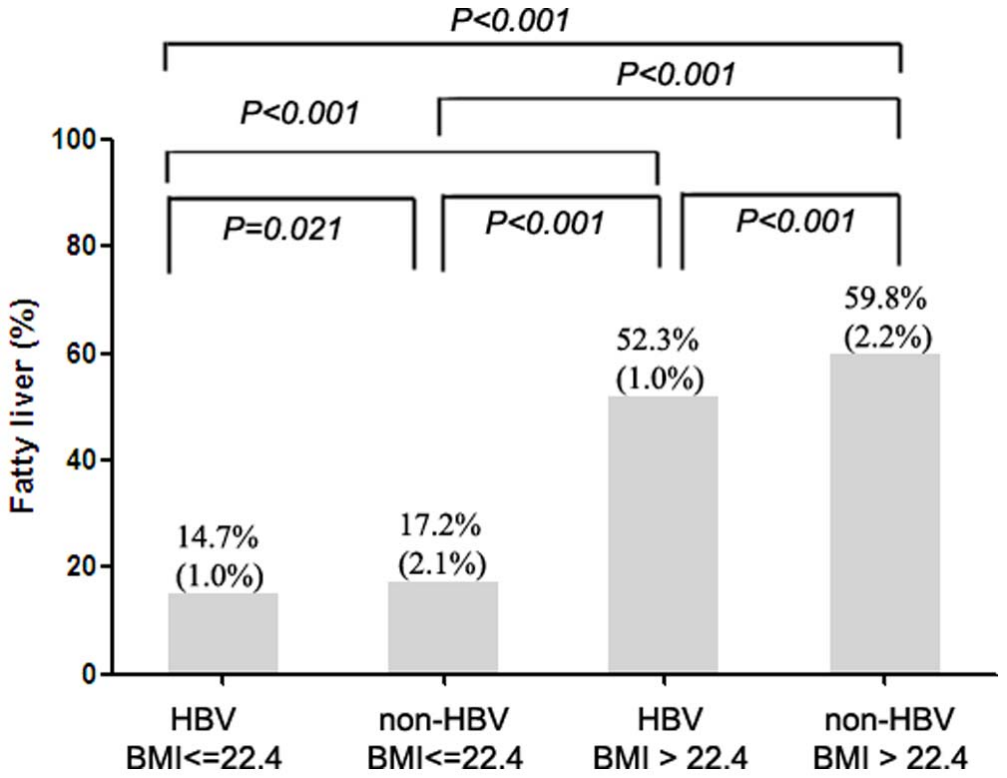
Distribution of fatty liver disease stratified by HBV status and BMI. The prevalence rates of fatty liver in all subgroups are expressed as prevalence rate (standard error). For subjects with BMI ≦22.4 kg/m^2^, the prevalence rate of fatty liver between those with and without HBV infection were insignificant by multivariate analysis though the P value was 0.021 by univariate analysis. For subjects with BMI >22.4 kg/m^2^ in combination with HBV infections have a significant lower rate of fatty liver (52.3%) in compared to those with BMI >22.4 kg/m^2^ but without HBV infections (59.8%).

For the whole subjects stratified by BMI, those with BMI ≦22.4 kg/m^2^ in combination with HBV infections have a lower rate of fatty liver (14.7%), comparing to those with BMI ≦22.4 kg/m^2^ and without HBV infections (17.2%). (Figure 3) However, the difference was not significant by multivariate analysis. (Table 4) On the contrary, subjects with BMI >22.4 kg/m^2^ in combination with HBV infections have a significant lower rate of fatty liver (52.3%) in compared to those with BMI >22.4 kg/m^2^ but without HBV infections (59.8%) both in univariate and multivariate analyses.

Stratified by age, BMI, sex, WC, fasting glucose, cholesterol, HDL, ALT, platelet count were significantly related with fatty liver whether patients were older or younger than 50 years. Nevertheless, viral status was not related to fatty liver in younger patients (age ≦50 years). On the contrary, the diagnosis of fatty liver was 0.624 fold less in HBV patients than in non-HBV patients with older than 50 years. (Table 4 and Figure 2).

### Factors Associated with Elevated Serum ALT Levels in Fatty Liver Stratified by HBV, BMI and Age

As shown in **Table 5**, for subjects with fatty liver, multivariate analysis showed that larger BMI, older age, higher WC, fasting glucose, cholesterol, HDL, higher GGT levels and HBsAg positivity were the independent risk factors correlated with elevated serum ALT levels. For subjects with HBV infection and fatty liver, younger age, larger WC, lower HDL, higher GGT, and platelet count were associated with elevated serum ALT levels by multivariate analysis.

**Table 5:** Multivariate analysis of factors associated with elevated serum ALT levels in subjects with fatty liver.

	Odds Ratio	95% Confidence interval	P
**All subjects**			
**BMI (per kg/m^2^)**	1.062	1.040–1.084	<0.001
**Age (per years)**	0.969	0.965–0.973	<0.001
**WC (per cm)**	1.023	1.014–1.031	<0.001
**Fasting glucose (per mg/dL)**	1.002	1.000–1.003	0.009
**Cholesterol (per mg/dL)**	1.004	1.002–1.005	<0.001
**HDL (per mg/dL)**	0.976	0.972–0.980	<0.001
**GGT (per IU/L)**	1.032	1.030–1.035	<0.001
**HBsAg positivity**	1.782	1.563–2.031	<0.001
**For subjects with HBV infection**			
**Age (per years)**	0.969	0.957–0.980	<0.001
**WC (per cm)**	1.022	1.007–1.037	0.005
**HDL (mg/dL)**	0.980	0.970–0.991	<0.001
**GGT (IU/L)**	1.038	1.030–1.046	<0.001
**Platelet (per 1000/mm^3^)**	0.980	0.970–0.991	<0.001
**For subjects with BMI≤22.4 kg/m^2^**			
**Age (per years)**	0.979	0.966–0.993	0.002
**Cholesterol (per mg/dL)**	1.008	1.004–1.011	<0.001
**HDL (per mg/dL)**	0.973	0.962–0.984	<0.001
**GGT (per IU/L)**	1.024	1.018–1.029	<0.001
**HBsAg positivity**	3.306	2.017–4.569	<0.001
**For subjects with BMI>22.4 kg/m^2^**			
**Age (per years)**	0.966	0.962–0.970	<0.001
**WC (per cm)**	1.036	1.030–1.042	<0.001
**Fasting glucose (per mg/dL)**	1.003	1.001–1.004	0.002
**Cholesterol (per mg/dL)**	1.003	1.002–1.004	<0.001
**HDL (per mg/dL)**	0.978	0.973–0.982	<0.001
**GGT (per IU/L)**	1.033	1.031–1.036	<0.001
**HBsAg positivity**	1.673	1.458–1.920	<0.001
**For subjects with age≦50 years**			
**BMI (per kg/m^2^)**	1.130	1.109–1.151	<0.001
**Cholesterol (per mg/dL)**	1.004	1.002–1.006	<0.001
**HDL (per mg/dL)**	0.978	0.971–0.984	<0.001
**GGT (per IU/L)**	1.032	1.029–1.036	<0.001
**HBsAg positivity**	1.681	1.400–2.017	<0.001
**For subjects with age >50 years**			
**BMI (per kg/m^2^)**	1.073	1.043–1.104	<0.001
**WC (per cm)**	1.014	1.004±1.025	0.008
**SBP (per mmHg)**	0.997	0.993–1.000	0.031
**Fasting glucose (per mg/dL)**	1.002	1.000–1.004	0.030
**Cholesterol (per mg/dL)**	1.004	1.002–1.005	<0.001
**HDL (per mg/dL)**	0.975	0.970–0.981	<0.001
**GGT (per IU/L)**	1.033	1.030–1.036	<0.001
**Platelet (per/mm^3^)**	0.998	0.997–0.999	<0.001
**HBsAg positivity**	1.946	1.614–2.347	<0.001

BMI, body mass index; WC, waist circumference; SBP, systolic blood pressure; HDL, high-density lipoprotein; ALT, alanine aminotransferase; GGT, gamma-glutamyltransferase; HBsAg, hepatitis B surface antigen.

For the whole subjects with fatty liver, HBsAg positivity, higher GGT levels, and presence of metabolic factors, such as higher cholesterol and lower HDL levels, were still associated with elevated serum ALT levels irrespective of age and BMI status. The present of elevated serum ALT levels were 3.306 fold, 1.673 fold, 1.681 fold and 1.946 fold more in HBV patients than in non-HBV patients with age ≦50 years, age >50 years, BMI≦22.4 kg/m^2^ and BMI >22.4 kg/m^2^, respectively. **(** **Table 5** **).**

## Discussion

The prevalence of NAFLD varies by the diagnostic modality and ethnicity and present in 23–51% in Asian populations [5], [6]. In the present study, fatty liver was diagnosed by ultrasonography in 43.9% of patients who received physical checkup in a single medical center. Of note, HBV infection was associated with lower prevalence of fatty liver, especially in subjects of overweight or obesity, or in those who were older than 50 years. However, the association between HBV and fatty liver was less apparent if patients were normal-weight or lean, or younger than 50 years. On the contrary, HBV infection was positively correlated with the presence of elevated liver biochemistries in subjects with fatty liver disease regardless of their age and BMI. It implies that viral factors might play some roles in the pathogenesis of fatty liver in subjects with fatty liver.

The most common cause of mortality in NAFLD patients is cardiovascular disease and the rate of cirrhosis development in patients with NAFLD is around 5–20% over 10 years [1], [22]. Identification and improvement of the risk factors of NAFLD may contribute to reduce these complications. The risk factors of fatty liver disease reported by previous studies include age, gender and metabolic factors, such as central obesity, higher BMI, elevated fasting blood glucose, TG, cholesterol and lower HDL levels [6], [23]. Our present study further validated that patients were at risk of fatty liver disease if they possessed these metabolic aberrances. While the evidence of all medications to improve NAFLD was inconclusive or conflicting, lifestyle interventions, such as diet modification, exercise programs and weight loss, and the treatment of the associated metabolic comorbidities such as obesity, hyperlipidemia, insulin resistance and type 2 diabetes mellitus are considered beneficial [1].

Fatty liver index, an algorithm based on BMI, waist circumference, triglycerides and GGT, was designed to assist the diagnosis of fatty liver and is used to predict patients at risk of type 2 diabetes mellitus and metabolic syndrome recently [24]. The present study demonstrated that FLI averaged 15.64 and 41.45 in non-fatty and fatty liver patients, respectively, with a significant difference.

The prevalence of fatty liver between HBV patients and general population was inconclusive. Nascimento et al. found that NAFLD presented in HBV patients from 10–76% of the time [25]. In Beijing, China, the frequency of fatty liver in HBV patients is higher than that for the general population, but lower than that in HCV patients [16]. On the contrary, the prevalence of NAFLD in HBV patients was less than that for the general population in Shanghai, China [26]. In our study, fatty liver was less in HBV patients (38.9%) than the general population (43.9%). Further stratification analysis showed that chronic HBV infection presented with no significant influence in the prevalence of fatty liver in patients younger than 50 years and the result was comparable with Wang et al.’s study whose mean age was 46.56 years [12]. Of note, the inverse relationships between the prevalence of fatty liver and positive HBsAg status was observed in patients older than 50 years. HBsAg seroclearance occurs more often after 50 years in patients with chronic hepatitis B [27]–[29], and Chu demonstrated that HBsAg carriers with moderate-to-severe hepatic steatosis were associated with more than threefold HBsAg seroclearance than those without hepatic steatosis [30]. These studies implied that there were inverse relationships between the prevalence of fatty liver and positive HBsAg status, especially in patients older than 50 years as demonstrated in our study [7], [15].

Higher BMI has been declared to be related with fatty liver in previous studies and it is consistent in the present study [1]. Further stratification analysis demonstrated that the presence of HBV infection was associated with less fatty liver in patients of BMI over 22.4 kg/m^2^. Obese patients with chronic HBV infection may be more concerned about their dietary habits and have less prevalence of fatty liver. Moreover, excessive BMI is involved in the transition from HBV carrier state to HCC and liver-related mortality [10], [31]. The reduced prevalence of fatty liver in HBV patients with higher BMI may also reflect the higher mortality in obese patients with chronic HBV infection [32]. As mentioned above, several studies have shown high HBsAg seroclearance in patients with hepatic steatosis [28]–[30]. Further stratification analysis in REVEAL-HBV study [18], [28], found that extreme obesity was associated with low HBV viral load and was a significant predictor of HBsAg seroclearance in chronic hepatitis B patients. This may be a reason why HBV patients with higher BMI had less fatty liver. Nevertheless, HBV infection was positively correlated with the elevated serum ALT levels in subjects with fatty liver disease regardless of their age and BMI, suggesting that liver inflammation induced by viral infection still played an important role for the formation and degree of NASH.

The strengths of this study are the large sample size and detailed biochemistry data. However, it has several limitations that are worth noting. First, the study population had a higher socioeconomic status and subjects could afford the expense of a physical check-up, so the results may not represent the general population. Second, some liver related diseases, such as alcohol consumption, medication use, autoimmune liver disease, and congenital liver disease, are not documented in the study. Nevertheless, the prevalence of elevated serum ALT levels due to alcohol consumption was low (0.8%) in a Taiwanese community study [33]. Drug-induced liver injury is rare and the incidence was 14 cases per 100,000 inhabitants per year in a French population-based prospective study [34], [35]. With available epidemiological data, autoimmune hepatitis, primary biliary cirrhosis, hematochromatosis and Wilson disease are all rare in Asia [36]–[38]. Those suggest that these factors may only play a small role and have little influence on the result. Third, smoking was not listed in the database though it has been noted as a risk for the progression of HBV and HCV infection [39]. Fourth, the gold standard for the diagnosis of fatty liver is liver biopsy. However, the invasiveness of that procedure is not justified for surveillance in the general population. In the present study, the diagnosis of fatty liver was made by ultrasonography and defined by at least two of three abnormal findings: diffusely increased echogenic liver greater than kidney or spleen, vascular blurring and deep attenuation of ultrasound signal with a sensitivity of 89% and specificity of 93% [40], [41]. Fifth, the rate of NAFLD progression to cirrhosis is about 5–20% over 10 years, which has a 10-year liver-related mortality of 25% [22]. However, our study is a cross-sectional design, so the prognosis of the population was unattainable. Sixth, severities of HBV infections, such as HBV viral load, hepatitis B e antigen status, HBV viral mutations, and the use of antiviral therapy, were not available in this study, which led to a difficulty of further analysis regarding these viral factors [42]–[44].

In conclusion, metabolic factors are imperative for the prevalence of fatty liver. Fatty liver disease is less common in HBV patients than in the general population, especially in older and obese patients. On the contrary, HBV infection was positively correlated with the presence of elevated liver biochemistries in subjects with fatty liver disease regardless of their age and BMI.
